# Safety Studies of Pneumococcal Endolysins Cpl-1 and Pal

**DOI:** 10.3390/v10110638

**Published:** 2018-11-15

**Authors:** Marek Harhala, Daniel C. Nelson, Paulina Miernikiewicz, Ryan D. Heselpoth, Beata Brzezicka, Joanna Majewska, Sara B. Linden, Xiaoran Shang, Aleksander Szymczak, Dorota Lecion, Karolina Marek-Bukowiec, Marlena Kłak, Bartosz Wojciechowicz, Karolina Lahutta, Andrzej Konieczny, Krystyna Dąbrowska

**Affiliations:** 1 Bacteriophage Laboratory, Institute of Immunology and Experimental Therapy, Polish Academy of Sciences, Rudolfa Weigla Street 12, 53-114 Wroclaw, Poland; marek.harhala@iitd.pan.wroc.pl (M.H.); pola@iitd.pan.wroc.pl (P.M.); beatabrzezicka94@gmail.com (B.B.); joanna.majewska@iitd.pan.wroc.pl (J.M.); szymczakaleksander@gmail.com (A.S.); dorota.lecion@wp.pl (D.L.); karolina.wojtyna@iitd.pan.wroc.pl (K.L.); 2 Institute for Bioscience and Biotechnology Research, University of Maryland, Rockville, MD 20850, USA; nelsond@umd.edu (D.C.N.); rheselpoth@rockefeller.edu (R.D.H.); slinden1@umd.edu (S.B.L.); sxr520@umd.edu (X.S.); 3 Research and Development Center, Regional Specialized Hospital, 51-124 Wrocław, Poland; marek-bukowiec@wssk.wroc.pl (K.M.-B.); klak@wssk.wroc.pl (M.K.); andrzej_konieczny@yahoo.com (A.K.); 4 Perlan Technologies Sp. z o. o., 02-785 Warsaw, Poland; bwojciechowicz@perlan.com.pl

**Keywords:** endolysin, Pal, Cpl-1, safety, toxicity, immune response, *Streptococcus pneumoniae*

## Abstract

Bacteriophage-derived endolysins have gained increasing attention as potent antimicrobial agents and numerous publications document the in vivo efficacy of these enzymes in various rodent models. However, little has been documented about their safety and toxicity profiles. Here, we present preclinical safety and toxicity data for two pneumococcal endolysins, Pal and Cpl-1. Microarray, and gene profiling was performed on human macrophages and pharyngeal cells exposed to 0.5 µM of each endolysin for six hours and no change in gene expression was noted. Likewise, in mice injected with 15 mg/kg of each endolysin, no physical or behavioral changes were noted, pro-inflammatory cytokine levels remained constant, and there were no significant changes in the fecal microbiome. Neither endolysin caused complement activation via the classic pathway, the alternative pathway, or the mannose-binding lectin pathway. In cellular response assays, IgG levels in mice exposed to Pal or Cpl-1 gradually increased for the first 30 days post exposure, but IgE levels never rose above baseline, suggesting that hypersensitivity or allergic reaction is unlikely. Collectively, the safety and toxicity profiles of Pal and Cpl-1 support further preclinical studies.

## 1. Introduction

*Streptococcus pneumoniae* is the most common cause of bacteremia, pneumonia, meningitis, and otitis media in children. Despite a successful vaccine campaign against pneumococcal disease over the past two decades, a Global Burden of Disease Study suggests that over 500,000 deaths still occur annually due to *S. pneumoniae* infection [1] and the World Health Organization estimated that 5% of all-cause mortality in children under five years of age is related to pneumococcal infection [2]. Furthermore, there has been a worldwide increase in antibiotic-resistant strains of *S. pneumoniae* [3,4], with high rates of resistance reported for penicillin (34.2%), trimethoprim-sulfamethoxazole (31.9%), and erythromycin (29.5%) [5]. The remarkable variability among *S. pneumoniae* strains further complicates vaccine approaches, with at least 92 capsular serotypes having been identified to date [6] and current conjugate vaccines only covering a small subset (i.e., 7–13 serovars) of the most common capsule serotypes. Therefore, alternative antimicrobial approaches to pneumococcal disease are highly desirable.

Bacteriophages, as viruses that infect and kill bacteria, use lytic proteins to destroy the bacterial membrane and peptidoglycan, resulting in lysis of the cell and the release of progeny phage. Among these proteins, endolysins are enzymes that function to break chemical bonds in the peptidoglycan. Appreciably, these enzymes can cause “lysis from without” on susceptible Gram-positive bacteria in the absence of phage due to their actions on the external cell wall and subsequent osmotic lysis of the unprotected bacterial membrane. Because of this unique property, endolysins have been shown to be highly effective antibacterial agents that represent an alternative to antibiotics [7,8,9].

Several pneumococcal-specific endolysins have been described in the literature. The two most notable of these endolysins are Pal, which is derived from the streptococcal Dp-1 phage [10], and Cpl-1, which is derived from the streptococcal Cp-1 phage [11]. These enzymes have been validated for efficacy in mouse pneumococcal bacteremia models [12,13]. Additionally, Pal and Cpl-1 have been validated in a mouse nasopharyngeal colonization model [14,15], and Cpl-1 has shown efficacy against *S. pneumoniae*-induced endocarditis in rats [16], meningitis in rats [17], and by aerosolized delivery in a mouse model of fatal pneumococcal pneumonia [18]. Lastly, the combinational use of Pal, which possesses an *N*-acetylmuramoyl-l-alanine amidase activity, and Cpl-1, which has an N-acetylmuramidase activity, displayed in vitro and in vivo synergistic efficacy [13,19].

Preclinical safety and toxicity profiles on mammalian cells and tissues are critically important for future translational development of endolysins. Here, we present a safety assessment of Pal and Cpl-1, including an in vivo assessment of safety in a mouse model and gene expression profiling to identify potential effects of these enzymes on human cell functions as well as non-cellular immunological complement cascades in vitro and ex vivo. Immune response assessment further includes inflammatory maker testing (i.e., IL-6) and endolysin-specific IgG and IgE antibody induction, with the latter providing an overall assessment of potential allergic reactions (type I hypersensitivity) [20,21].

## 2. Materials and Methods

### 2.1. Protein Expression

Two endolysins—Pal (Acc. no. YP_004306947) from *Streptococcus* phage Dp-1, and Cpl-1 (Acc. no. CAA87744) from *Streptococcus* phage Cp-1—were investigated. The genes for Cpl-1 and Pal were originally amplified directly from the Cp-1 phage and the Dp-1 phage, respectively, into the Gateway cloning system. Subsequently, primers incorporating a C-terminal 6×-His tag were used to amplify from template plasmids containing the native genes. The resulting amplicons were cloned into a pBAD24 expression plasmid. Vectors were expressed in *E. coli* B834(DE3) cells (EMD), and grown at 37 °C with shaking in Luria-Bertani (LB) broth, supplemented with 10 g/L NaCl and ampicillin (100 µg/mL) (all from Sigma-Aldrich, Europe), until OD_600_ reached 1.0. Then, the bacterial culture was cooled to 20 °C and protein expression was induced by the addition of arabinose at a final concentration of 2 g/L. The culture was subsequently incubated overnight at 20 °C with aeration by shaking.

### 2.2. Protein Purification

Bacteria were harvested using centrifugation and suspended in phosphate buffer (50 mM Na_2_HPO_4_, 300 mM NaCl, pH 8), which was supplemented with PMSF (1 mM) and lysozyme (0.5 mg/mL). The slurry was incubated for 6–7 h on ice and lysed using the freeze-thaw method. Mg^2+^ (up to 0.25 mM), DNAse (10 μg/mL), and RNAse (20 μg/mL) were then added to the extract and allowed to incubate on ice for an additional 3 h. The fractions were separated using centrifugation (12,000× g, 45 min, 4 °C) and the soluble fraction (supernatant) was collected. The samples were then incubated with NiNTA agarose (Qiagen, Hilden, Germany) at room temperature for 10 min and washed with PBS (5× volume of the agarose) and an increasing concentration of imidazole (0 mM, 20 mM, 100 mM, 250 mM, and 500 mM). The 100 mM and 250 mM fractions containing the eluted endolysins were dialyzed against PBS at 4 °C. Next, proteins were separated using gel filtration (fast protein liquid chromatography) on a Superdex 75 10/300 GL column (GE Healthcare Life Sciences, Chicago, IL, USA). The final step was LPS removal, which was performed with an EndoTrap Blue column (Hyglos GmbH, Munich, Germany). Purified, endotoxin-free protein samples were dialyzed against PBS and filtered through sterile 0.22-μm polyvinylidene difluoride filters (Millipore, Burlington, MA, USA). All the purification steps were monitored using SDS-PAGE electrophoresis and the LPS (endotoxin) content was determined using the EndoLISA assay (Hyglos GmbH, Munich, Germany) and confirmed to be 0.6–1.6 endotoxin units (EU) per mL for all of the microarray gene expression profiling and less than 10 EU per animal in animal experiments.

### 2.3. Microarray Gene Expression Profiling

The cell lines FaDu (human pharynx squamous cell carcinoma, ATCC HTB-43) and SC (human peripheral blood macrophages, ATCC CRL-9855) were cultured in media recommended by the manufacturer with and without protein solutions containing Pal or Cpl-1 (0.5 µM, i.e., 17.5 and 20 µg/mL, respectively) for 6 h. This time was chosen to allow for the development of cellular responses to external factors at the cellular gene expression level. Control cells were cultured with an equivalent amount of albumin (Sigma, Poznan, Poland). After incubation, cells were harvested and the total RNA was immediately isolated with RNeasy Mini Kit (Qiagen). For this study, we used SurePrint G3 Human Gene Expression v3 8 × 60 K Microarrays (Agilent Technologies, Santa Clara, CA, USA). The One-Color Microarray-Based Gene Expression Analysis Protocol (version 6.9.1) was used to process the arrays.

After amplification, the total RNA was labeled with Cy3 using the Low Input Quick Amp Labeling Kit (Agilent Technologies, USA). The labeled RNA was purified (RNeasy Kit, Qiagen, USA), and the RNA yield (nanograms of complementary RNA (cRNA)) as well as specific activity (picomoles of Cy3 per microgram of cRNA) were measured using a NanoQuant plate (Tecan Group, Germany) in an Infinite 200 PRO reader. Next, the labeled cRNA was fragmented and placed on the microarray slide after mixing with the hybridization buffer. Microarrays were incubated for 17 h at 65 °C and then washed twice in GE wash buffer (Agilent). Agilent’s High-Resolution C Microarray Scanner was used to scan the slides according to the 8 × 60 K array format. The scanned images were analyzed with the Agilent Feature Extraction software v. 12.1 (Agilent Technologies, Santa Clara, CA, USA). The final analysis included dye normalization (linear and LOWESS), background subtractions, and filtering of outlier spots.

#### 2.3.1. Differentially Expressed Genes

GeneSpring GX 13 (Agilent, USA) was used to further analyze the data after extraction. The cut-off was set to 1.5-fold for the determination of significant differential expression (up or down regulation). A moderated Student’s *t* test was used to determine significant differences, defined as *p* ≤ 0.05, for gene expression.

#### 2.3.2. Enriched Gene Ontology Terms & Pathway Analysis

Lists of differentially expressed genes were uploaded to the DAVID 6.7—Database for Annotation, Visualization and Integrated Discovery Classification System to analyze their ontologies and participation in curated pathways, and to perform a Gene Set Enrichment Analysis (GSEA). Ontologies and pathways were assigned independently to upregulated and downregulated gene lists. Annotations (official gene symbols) were limited to *Homo sapiens* and the genome of this species was used as a background for analyses. For the pathway analysis, a Kyoto Encyclopedia of Genes and Genomes (KEGG) was used as a reference database.

### 2.4. Animal Experiments

Six- to twelve-week-old male mice, either C57BL6/J (*N* = 6) or BALB/c (*N* = 5 or 7), were bred at the Animal Breeding Centre of the Institute of Immunology and Experimental Therapy (IIET). All the animal experiments were approved by the 1st Local Committee for Experiments with the Use of Laboratory Animals, Wrocław, Poland (projects no. 76/2011) and performed according to EU directive 2010/63/EU. All the animal experiments adhered to the ARRIVE (Animal Research: Reporting of In Vivo Experiments) guidelines [22].

For the general health condition (Section 2.5), the microbiome assessment (Section 2.6), and the inflammatory/cytokine assessment (Section 2.7), BALB/c mice were injected with Pal or Cpl-1 intraperitoneally in one dose at 0.3 mg per mouse (15 mg/kg) for each protein. The negative control was inoculated with PBS and a positive control of inflammation was inoculated with LPS (2000 EU/mouse). Murine blood was collected into clotting tubes under anesthesia from the tail vein. Serum was separated from the blood using double centrifugation (2250× *g* for 5 min and 10,000× *g* for 10 min). The sera were stored at −20 °C.

### 2.5. Overall Health Scoring Matrix of Mice

A composite scoring matrix indicating the general health of mice was calculated on a 15-point scale by adding the scores of five aspects, each rated from 0 to 3 (with mid-values possible), where the highest score represented the worst condition. The specific criteria used included: Activity (0 = alert and active, or calm and resting; 3 = slow to move or non-responsive when coaxed or violent reaction to stimuli); Fur (0 = normal, well groomed; 3 = very rough hair coat); Eyes (0 = normal, open, clean, no exudate; 3 = closed, sunken or covered with suppurate exudates); Abdomen (0 = normal; 3 = large abdominal mass and/or edema); and Skin (0 = normal, healthy skin; 3 = wounds, dermatitis, lesions).

### 2.6. Microbiome Assessment of Mice

Mice were injected *i.p*. with Pal, Cpl-1, or PBS (control), as described in Section 2.4, and fresh fecal samples were analyzed prior to treatment or 24 h after treatment. DNA isolation from mice feces was performed with a QIAamp DNA Stool Mini Kit preceded by physical homogenization with 0.1 mm zirconia beads. A total of 1 mL of InhibitEX buffer was added to the tubes containing the beads and stool samples. The samples were vortexed for 1 min and then incubated at 95 °C for 5 min. Next, each sample was vigorously vortexed for 3 min and centrifuged for 1 min at 14,500 rpm. Further isolation was performed according to the manufacturer’s instructions with additional cleaning utilizing the Zymo Clean & Concentrator. The DNA was eluted with deionized water and the samples were stored at –20 °C for further use.

A preliminary measurement of the isolated DNA was performed with the Qubit 2.0 fluorometer using the Qubit™ dsDNA HS assay kit (Life Technologies Corp., Eugene, OR, USA). Three samples of the highest quality from each group were selected for further processing. The Ion 16S Metagenomics Kit (ThermoFisher, Waltham, MA, USA) was used to amplify DNA coding for the 16S rRNA V2, V3, V4, V5, V6–7, V8, and V9 hypervariable regions, according to the manufacturer’s instructions using 5 ng of DNA for each sample. Barcoded libraries were created using the Ion Xpress™ Plus Fragment Library Kit with the Ion Xpress Barcodes. The final library concentration was quantified by RT-qPCR with the Ion Library TaqMan Quantitation Kit according to manufacturer’s protocol. Emulsion PCR and a bead enrichment step were performed on an Ion OneTouch™ 2 System with the Ion PGM Hi-Q View OT2 kit. Enriched template beads were mixed with reagents from the Hi-Q View 400 Sequencing kit and loaded onto Ion Torrent 314 V2 chips. Sequencing parameters standard for 16S rRNA Targeted Sequencing were used based on the manufacturer’s protocol.

Unaligned binary data files (Binary Alignment Map (.BAM)) generated by the Ion Torrent PGM were uploaded to IonReporter version 5.6. An analysis was performed with the base pair cut-off number set at 150, minimum alignment coverage at 90%, and minimum abundance at 10 copies. Curated MicroSEQ 16S Reference Library v2013.1 was used as a reference database to identify the reads obtained. The results received by the workflow described were visualized using KRONA software integrated into IonReporter 5.6.

### 2.7. Cytokine Assay

The progression of the inflammatory reaction in the murine blood was monitored by measuring interleukin-6 (IL-6) serum levels using a commercially available Human/Mouse IL-6 Mini ABTS ELISA Development Kit (PeproTech Inc., Rocky Hill, NJ, USA) following the recommendations of the manufacturer.

### 2.8. Specific Sera Induction

For specific IgG induction, C57BL6/J mice were challenged *s.c.* with Pal or Cpl-1 (0.05 mg per mouse) on day 0. Murine blood was collected into clotting tubes under anesthesia from the tail vein every 3–5 days. Serum was separated from the blood using double centrifugation (2250× *g* for 5 min and 10,000× *g* for 10 min). The sera were stored at −20 °C.

### 2.9. Measurement of Specific IgG and IgE Antibody Levels

MaxiSorp flat-bottom 96-well plates (Nunc, Thermo Scientific, Waltham, MA, USA) were sterilely coated overnight at 4 °C with endolysins or PBS as control using 100 μL per well at a concentration of 10 μg/mL. Subsequently, wells were washed 5 times with PBS and blocked for 1 h with 1% albumin or SuperBlock Blocking buffer (Life Technologies Europe BV, Bleiswijk, The Netherlands) at 150 μL per well at room temperature. Solution was removed and the plates were washed 5 times with 0.05% Tween 20 (AppliChem GmbH, Darmstadt, Germany) in PBS at 100 μL per well. One hundred microliters per well of diluted serum (1:100 in PBS) was applied to the wells coated with endolysins as well as control wells. Each sample was investigated in duplicate. The plates were incubated at 37 °C for 2 h after which serum was removed and the plates were washed 5 times with 0.05% Tween 20 in PBS at 100 μL per well. One hundred microliters per well of diluted detection antibody (peroxidase-conjugated goat anti-mouse IgG (Jackson ImmunoResearch Laboratories, Cambridgeshire, UK) or IgE (ThermoFisher, Waltham, MA, USA)) was applied to the plates and incubated for 1 h at room temperature in the dark. The antibody solution was removed and the plates were washed with PBS with 0.05% Tween 20 5 times at 100 μL per well. TMB (50 μL) was used as a substrate reagent for peroxidase according to the manufacturer’s instructions (R&D Systems, Minneapolis, MN, USA) and incubated for 30 min. Twenty-five microliters of 2N H_2_SO_4_ was added to stop the reaction and the absorbance was measured at 450 nm (main reading) and normalized by subtracting the background absorbance at 570 nm.

As a reference level of specific IgE antibody induction, an oral mouse allergy model to ovalbumin (OVA) was utilized. Allergy to OVA was induced in mice using a dedicated adjuvant as described [23]. Briefly, the mice were injected subcutaneously with OVA (50 µg/mouse) with Al(OH)_3_ as an adjuvant promoting the hypersensitivity reaction. The injection was repeated after 14 days. Seven days after the second sensitization, the mice were given 20% Egg White Solution (EWS) (Sigma, Poznan, Poland) in the drinking water. IgE levels specific for OVA were evaluated using ELISA as described above and compared to control mice injected with PBS instead of OVA.

### 2.10. Complement System Activity Test

Complement assays were performed with the WIESLAB^®^ Complement System kits (Euro Diagnostica, Lundavagen, Sweden) to test the activation of the three complement pathways in the presence of Pal or Cpl-1. Briefly, the kits contained pre-coated plates with specific activators for the classic pathway (CP), alternative pathway (AP), or mannose-binding lectin (MBL) pathway (LP). Blood samples were collected from six healthy human donors. Blood was collected in clotting tubes and incubated for 60 min at room temperature, followed by 10 min centrifugation at 2000× *g*, and isolated serum was immediately used for the tests. First, human serum was incubated at 37 °C for 10 min with 2 µg/mL of Pal, Cpl-1, or PBS in equal volumes. Then, sera were diluted with the provided buffers at 1: 200, 1:18, 1:100 for the CP, AP and LP pathways, respectively, and transferred to the WIESLAB plate and processed according the manufacturer’s guidelines. Positive and negative controls included in the test kit served as quality control of the assay.

### 2.11. Research Ethics Involving Human Subjects

All the subjects gave their informed consent for inclusion before they participated in the study. The study was conducted in accordance with the Declaration of Helsinki, and the protocol was approved by the Bioethics Committee of Regional Specialist Hospital in Wrocław (Project identification code: KB/nr 2/2017).

## 3. Results

### 3.1. Microarray Gene Expression Profiling in Human Cells Exposed to Pal or Cpl-1

Analysis of gene expression profiles in eukaryotic cells and their potential changes after exposure to investigated agents provides a sensitive tool for the detection of effects that those agents exert in eukaryotic cells, including potential toxicity and harmful effects. Therefore, gene expression patterns were tested in two human cell lines, normal SC macrophages and a pharyngeal carcinoma cell line, FaDu. Cell lines were exposed to 0.5 µM Pal or Cpl-1 (17.5 and 20 µg/mL, respectively), or albumin (BSA) as a control, for 6 h. DNA microarray analysis (SurePrint G3 Human Gene Expression v3 8 × 60 K Microarrays; Agilent Technologies, USA) showed no statistically significant (*p* < 0.05) changes in gene expression in either cell line when exposed to endolysins using two different types of statistical analysis, the Bonferroni Holm method for the family-wise error rate (FWER) and the Benjamini Hochberg method for the false discovery rate (FDR) (Supplementary Tables S1 and S2). Taken together, these results demonstrate that Pal and Cpl-1 had no negative effects on the human cells, including provocation of a toxicity or inflammatory response.

### 3.2. Effect of Pal and Cpl-1 on Complement System Activity in Human Blood Ex Vivo

**Figure 1:**
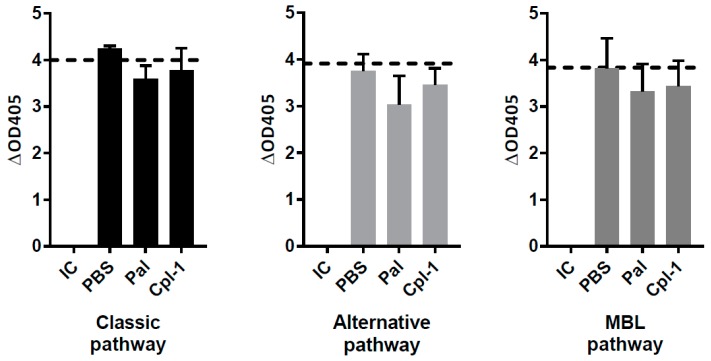
Ex vivo activity of the complement system in human serum incubated with Cpl-1, Pal or PBS. Pal and Cpl-1 tested at a concentration of 2 µg/mL. IC: control of inactivated complement system (as supplied by the manufacturer), dash line—control of normal complement activity (as supplied by the manufacturer).

The complement system is a non-cellular component of blood that plays an important role in immune responses. Therefore, in addition to the cellular assays, potential effects of endolysins on the complement system were also measured ex vivo in human blood with or without Cpl-1 and Pal. Diagnostic complement activity tests included those for the classic, alternative, and MBL-dependent pathways. As can be seen in Figure 1, neither Cpl-1 nor Pal had any effect on the activation of the complement system compared to the control. These results demonstrate that Cpl-1 and Pal do not activate the first line of non-cellular immune response in humans.

### 3.3. Overall Health Condition, Inflammation, and Microbiome Assessment in Mice Challenged with Pal or Cpl-1

**Figure 2:**
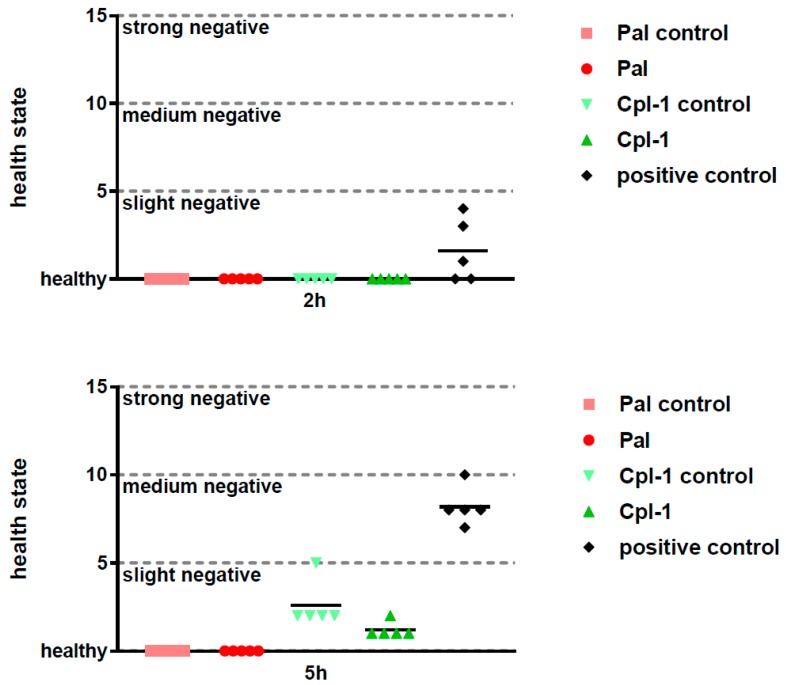
Composite health matrix of mice treated intraperitoneally with Pal and Cpl-1. Pal: mice treated with 0.3 mg of Pal; Pal control: mice treated with PBS containing the same residual LPS content as Pal (0.6 EU); Cpl-1: mice treated with 0.3 mg of Cpl-1; Cpl-1 control: mice treated with PBS containing the same residual LPS content as Cpl-1 (8 EU); positive control: mice treated with a toxic LPS dose (2000 EU). Upper panel: 2 h after treatment; Lower panel: 5 h after treatment.

**Figure 3:**
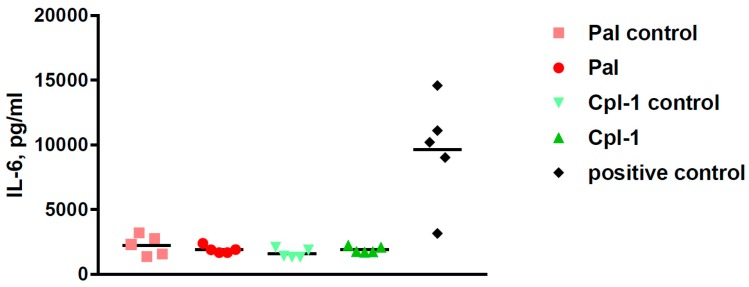
IL-6 cytokine levels in murine blood 5 h after intraperitoneal treatment with Pal or Cpl-1. Pal: mice treated with 0.3 mg of Pal; Pal control: mice treated with PBS containing the same residual LPS content as Pal (0.6 EU); Cpl-1: mice treated with 0.3 mg of Cpl-1; Cpl-1 control: mice treated with PBS containing the same residual LPS content as Cpl-1 (8 EU); positive control: mice treated with a toxic LPS dose (2000 EU).

Side effects of potential therapeutics may be demonstrated in vivo even though the in vitro and ex vivo assays suggest relative safety of the investigated therapeutics. To this end, we assessed the overall health condition of mice treated with Pal or Cpl-1, as well as the levels of the inflammatory marker, interleukin 6 (IL-6). The composite health status of the mice was assessed and scored 2 h and 5 h after injection with Pal and Cpl-1. Negative controls were treated with the same level of LPS as contained in the purified endolysin solutions and a toxic dose of LPS (2000 EU per mouse) served as a positive control of toxicity. Neither Pal nor Cpl-1 displayed any negative effects on the composite health status of mice compared to the negative controls (Figure 2). In contrast, the mice that received the high LPS dose had a marked, negative effect on their scoring matrix at both time points. A potential inflammatory response to Pal or Cpl-1 was further assessed using measurements of IL-6 levels in murine blood 5 hours after treatment with the endolysins. No significant differences were noted between Pal- or Cpl-1-treated mice compared to control mice for IL-6 production, yet a significant response was seen with mice given a high LPS dose (Figure 3), which recapitulates the health scoring results.

Because endolysins specifically destroy bacterial cells, their therapeutic use can potentially affect the microbiome of living individuals similar to the actions of traditional antibiotics. Abnormalities of microbiome composition can, in turn, affect the overall health status of an individual. Therefore, in mice treated *i.p*. with Pal or Cpl-1, the fecal bacterial microbiome composition was assessed using 16S rRNA targeted sequencing. Analysis of microbiome before treatment and 24 h after treatment revealed no meaningful changes in the general composition of bacterial groups (Supplementary Table S3, Figures S1 and S2). Specifically, the major groups remained unchanged (and no loss was observed in the Firmicutes group, which contains bacteria sensitive to Pal and Cpl-1).

### 3.4. Antibody Induction in Mice Challenged with Pal and Cpl-1

**Figure 4:**
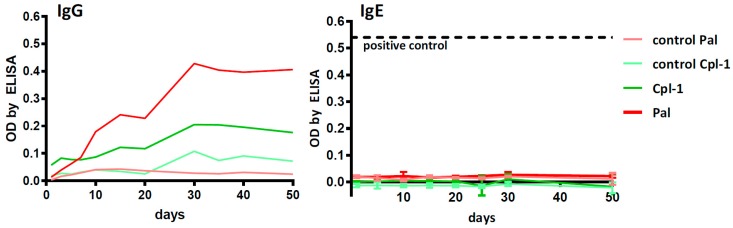
Specific IgG and IgE antibody levels in mice challenged with Pal or Cpl-1. Mice (*N* = 6) were injected intraperitoneally with enzymes 50 µg per mouse and control animals were injected with the relevant amount of LPS. Serum samples were collected on days 1, 5, 10, 15, 20, 25, 30, and 50 and tested using ELISA with Pal or Cpl-1 as the antigens. Pal: Pal-specific antibody level in mice treated with Pal; control Pal: Pal-specific antibody level in control mice; Cpl-1: Cpl-1-specific antibody level in mice treated with Cpl-1; control Cpl-1: Cpl-1-specific antibody level in control mice. Positive control reference level in IgE: antibodies specific to a model antigen (OVA) in allergic mice (Supplementary Figure S3).

Specific immune responses to Pal and Cpl-1 were assessed in a mouse model through the analysis of specific antibody induction. The serum levels of IgG were measured to evaluate the normal antibody response and serum IgE levels were determined for a potential hypersensitivity response to the endolysins. Murine sera were collected at days 1, 5, 10, 15, 20, 25, 30 and 50 after a single-dose application of each enzyme. As expected, a typical pattern of IgG induction was observed, with a slow increase in the specific antibody titer until ~day 30, then the titers leveled off (Figure 4). Importantly, during the entire 50-day evaluation period, no increase was observed for Pal- or Cpl-1-specific IgE relative to control mice (*p* < 0.05) (Figure 4). As a control to demonstrate the induction of IgE hypersensitivity, the same assays were performed on mice sensitized to OVA, which showed a large IgE response after 21 days (Supplemental Figure S3). Thus, we detected no propensity for the development of an IgE-mediated allergic reaction to Pal or Cpl-1.

## 4. Discussion

Bacteriophage endolysins have received increasing attention as potent alternatives to traditional antibiotics in the past decade and at least three companies are enrolling patients for Phase 2 clinical trials with endolysin-based therapeutics, according to clinicaltrials.gov. Nonetheless, there are few published articles describing the safety or toxicity of endolysins. The exception is SAL200, a pharmaceutical composition containing the SAL-1 endolysin, which targets *Staphylococcus aureus*. SAL200 showed no toxicity in rodent and dog single- and repeated-dose studies and pharmacology studies demonstrated no signs of toxicity in central nervous and respiratory system function tests [24]. Next, SAL200 was tested in monkeys to obtain pharmacokinetic and safety information. No laboratory abnormalities or adverse events were detected after injection of a single dose (up to 80 mg/kg per day) or injection of multiple doses (up to 40 mg/kg per day) [25]. Finally, the results for the human Phase 1 study of SAL200, the first in-human study for a bacteriophage endolysin-based drug, were recently published [26]. SAL200 was well-tolerated, and no serious adverse events were observed in this clinical study, with most adverse events being mild, self-limiting, and transient. Furthermore, no clinically significant values with respect to clinical chemistry, hematology, coagulation, urinalysis, vital signs, or physical examinations were observed.

In the present study, two pneumococcal endolysins, Pal and Cpl-1, were investigated for their potential effect on mammalian cells, cellular and non-cellular immune responses, microbiome changes, inflammatory response, and overall safety in a single-dose study in mice. We found that in most cases, Pal and Cpl-1 were neutral and exerted minor or no effect on cell or systemic functions.

Microarray and gene profiling analyses of human SC macrophages and human pharyngeal FaDu cells were analyzed after exposure to Pal and Cpl-1. Macrophages were chosen as they allow for the identification of potential effects of the endolysins on immune responses, whereas the pharyngeal cells represent non-immunological cells from a body site commonly colonized by *S. pneumoniae* [27,28]. In both types of cells, no statistically significant changes in the expression levels of over 16,000 genes were noted compared to albumin-treated cells (Tables S1 and S2), indicating that no specific pathways, like apoptosis or inflammatory responses, were activated by the enzymes, thus strongly supporting the safety of Pal and Cpl-1. It should be noted that Entenza et al. found increased serum pro-inflammatory cytokine (i.e., IL-1β, IL-6, TNF-α, and INF-γ) levels in a rat endocarditis model of *S. pneumoniae* when treated with high, continuous levels of Cpl-1 [16]. However, both our gene profiling results as well as the direct serum measurements of IL-6 (Figure 3) showed no increase due to endolysins, suggesting that the results of Entenza et al. reflect responses to the lysed bacterial cells rather than endolysins themselves. This is supported by Witzenrath et al., who showed that lower levels of Cpl-1 reduced pro-inflammatory cytokines in infected mice relative to untreated, infected mice [29]. It was hypothesized that higher concentrations of Cpl-1 generate a more complete digestion of the peptidoglycan, thereby generating more pro-inflammatory fragments to induce a response. Due to the lytic nature of these enzymes, detailed dosing studies in conjunction with safety profiles will be needed to achieve an optimal safe, effective dose. Nonetheless, our results demonstrate that the purified enzymes themselves cause no inflammatory responses.

Our results show that an IgG response is generated toward endolysins, which is not unexpected due to the proteinaceous nature of these enzymes. However, it has been reported that antibodies do not neutralize the catalytic activity of endolysins. Specifically, hyperimmune serum raised against Cpl-1 only slowed the killing kinetics of Cpl-1 against *S. pneumoniae* in vitro compared to identical experiments using pre-immune serum [12] (Figure S4). Likewise, Cpl-1 equally reduced the pneumococcal titer in the blood of both naïve mice and mice previously vaccinated with Cpl-1 in a bacteremia model. Similar results demonstrating high titer, but non-neutralizing effects of antibodies directed against endolysins have been reported for the MV-L and ClyS staphylococcal endolysins [30,31]. At present, it is not known why antibodies do not neutralize the activity of endolysins, but it is known that endolysins bind epitopes on the bacterial surface with nanomolar to picomolar affinities [32,33], which could out-compete the binding of the antibodies. The accumulated results suggest that endolysins, including Pal and Cpl-1, may be used as therapeutics repeatedly without diminished activity due to antibody response. Moreover, our results demonstrate for the first time in any pneumococcal endolysin that Pal and Cpl-1 do not generate an IgE response associated with hypersensitivity and allergic reaction.

One advantage of endolysin therapy is the exquisite specificity, often confined to a single species, displayed by the endolysins [8,9]. The targeted killing by endolysins prevents collateral bacteriolytic effects on commensal organisms and dysbiosis often associated with conventional antibiotic therapy. In the case of Pal and Cpl-1, the pneumococcal specificity is defined by C-terminal choline binding domains in each enzyme that bind with high affinity to the choline-containing wall teichoic acids of *S. pneumoniae* [34]. This is an advantage of Cpl-1 and Pal over other endolysins; only a few related oral bacteria from the mitis group are poorly susceptible compared to the rapid bactericidal activity on pneumococci. In this line of evidence, it is unlikely that Cpl-1 or Pal would have substantial bactericidal activity on the bacteria present in the fecal microbiome. As such, changes in the microbiome were not anticipated, and indeed were not observed (Supplementary Table S2, Figure S2). Cheng et al. showed that low doses (5 µg) of LysEF-P10 did not affect the gut microbiome, but that high doses (100 µg) did affect the microbiome, specifically lowering members of the geneus *Enterococcus* [35]. In contrast, our results showed that 300 µg of either Cpl-1 or Pal had no effect on the fecal microbiome. This is likely attributable to either the broad specificity of LysEF-P10 or the natural abundance of the enterococci in the fecal microbiome compared to the very narrow specificity of Cpl-1/Pal and the low natural abundance of pneumococci in the fecal microbiome.

We also tested the safety of both endolysins in vivo, at concentrations (0.3 mg/mouse; ~15 mg/kg based on a 20 g mouse) higher than previously used to show the efficacy of Cpl-1 in a pneumococcal sepsis model [13]. The overall composite health score indicated no adverse effects of endolysin treatment (Figure 2). Only the Cpl-1 and the Cpl-1 control showed any signs that deviated from perfect health, although the signs did not even reach the level of a slight effect. Additionally, the endotoxin levels in the Cpl-1 preparations was >10 times higher than the levels in the Pal preparations (8 EU vs. 0.6 EU), underscoring the importance of endotoxin removal and testing. Taken together, our preclinical safety results of Pal and Cpl-1 support the implementation of future escalating-dose safety studies, as well as pharmacokinetic and pharmacodynamic studies in larger mammals and primates.

## Supplementary Materials

The following are available online at http://www.mdpi.com/1999-4915/10/11/638/s1, Table S1: Statistical data from the analysis of gene expression in SC cells exposed to Pal or Cpl-1 compared to albumin (Alb), Table S2: Statistical data from the analysis of gene expression in FaDu cells exposed to Pal or Cpl-1 compared to albumin (Alb), Table S3: Composition of bacterial microbiome component in mice after treatment with Pal or Cpl-1, Figure S1: Shannon and Simpson indexes for bacterial microbiome in the gut of mice challenged with Pal or Cpl-1, Figure S2: Composition of bacterial microbiome component in mice before and after treatment with Pal or Cpl-1, Figure S3: Serum levels of IgE specific for OVA, positive reference of allergy in mice. Figure S4: Bacterial culture density 15 min after treatment with Cpl-1 or Pal lysins (final concentration: 25 μg/mL).
