# Supplementary material for: Safety Studies of Pneumococcal Endolysins Cpl-1 and Pal
Source: Viruses. 2018 Nov 15;10(11):638. doi: 10.3390/v10110638 (PMC6266847; doi:10.3390/v10110638)
Supplement: Supplementary file 1 [file viruses-10-00638-s001.zip › Supplementary/Figure_S2.pdf]

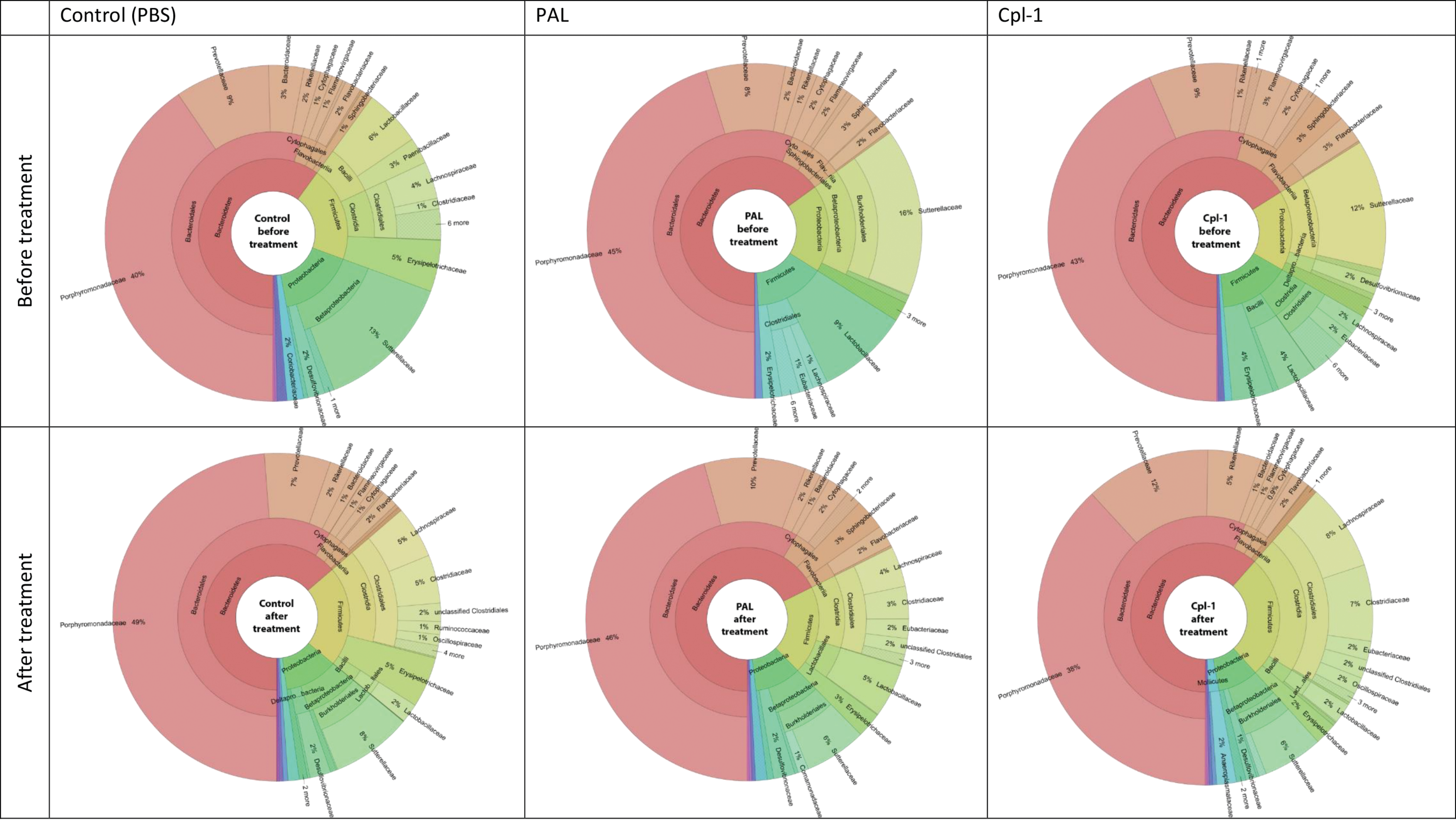

Figure S2. Composition of bacterial microbiome component in mice before and after treatment with Pal and Cpl-1.
