# Supplementary material for: Safety Studies of Pneumococcal Endolysins Cpl-1 and Pal
Source: Viruses. 2018 Nov 15;10(11):638. doi: 10.3390/v10110638 (PMC6266847; doi:10.3390/v10110638)
Supplement: Supplementary file 1 [file viruses-10-00638-s001.zip › Supplementary/Table_S2.pdf]

Test Description

Selected Test:	Moderated T-Test
Entity List:	Filtered on Flags [Detected, Not Dete...
Interpretation:	PAL vs. Alb
p-value computation:	Asymptotic
Multiple Testing Correction:	Bonferroni-Holm

Result Summary

	P all	P < 0.05	P < 0.02	P < 0.01	P < 0.005	P < 0.001
FC all	58155	0	0	0	0	0
FC > 1.1	42190	0	0	0	0	0
FC > 1.5	6884	0	0	0	0	0
FC > 2.0	1824	0	0	0	0	0
FC > 3.0	248	0	0	0	0	0
Expecte...		2907	1163	581	290	58

Test Description

Selected Test:	Moderated T-Test
Entity List:	Filtered on Flags [Detected, Not Detect...
Interpretation:	PAL vs. Alb
p-value computation:	Asymptotic
Multiple Testing Correction:	Benjamini-Hochberg

Result Summary

	P all	P < 0.05	P < 0.02	P < 0.01	P < 0.005	P < 0.001
FC all	58155	0	0	0	0	0
FC > 1.1	42190	0	0	0	0	0
FC > 1.5	6884	0	0	0	0	0
FC > 2.0	1824	0	0	0	0	0
FC > 3.0	248	0	0	0	0	0
Expecte...		0	0	0	0	0

Test Description

Selected Test:	Moderated T-Test
Entity List:	Filtered on Flags [Detected, Not Dete...
Interpretation:	Cpl-1 vs. Alb
p-value computation:	Asymptotic
Multiple Testing Correction:	Bonferroni-Holm

Result Summary

	P all	P < 0.05	P < 0.02	P < 0.01	P < 0.005	P < 0.001
FC all	58155	0	0	0	0	0
FC > 1.1	40630	0	0	0	0	0
FC > 1.5	6795	0	0	0	0	0
FC > 2.0	2025	0	0	0	0	0
FC > 3.0	323	0	0	0	0	0
Expecte...		2907	1163	581	290	58

Test Description

Selected Test:	Moderated T-Test
Entity List:	Filtered on Flags [Detected, Not Detec...
Interpretation:	Cpl-1 vs. Alb
p-value computation:	Asymptotic
Multiple Testing Correction:	Benjamini-Hochberg

Result Summary

	P all	P < 0.05	P < 0.02	P < 0.01	P < 0.005	P < 0.001
FC all	58155	0	0	0	0	0
FC > 1.1	40630	0	0	0	0	0
FC > 1.5	6795	0	0	0	0	0
FC > 2.0	2025	0	0	0	0	0
FC > 3.0	323	0	0	0	0	0
Expecte...		0	0	0	0	0
