# Supplementary material for: Safety Studies of Pneumococcal Endolysins Cpl-1 and Pal
Source: Viruses. 2018 Nov 15;10(11):638. doi: 10.3390/v10110638 (PMC6266847; doi:10.3390/v10110638)
Supplement: Supplementary file 1 [file viruses-10-00638-s001.zip › Supplementary/Table_S3.pdf]

Table S3: **Composition of bacterial microbiome component in mice before and after treatment with PAL and Cpl-1.**

	Before treatment				After treatment			
	Bacteroides	Firmicutes	Proteobacteria	Others	Bacteroides	Firmicutes	Proteobacteria	Others
**Cpl-1**	66 %	15 %	17 %	1.5 %	62 %	27 %	9 %	2.5 %
**PAL**	65 %	15 %	19 %	0.8 %	68 %	20 %	10 %	2.0 %
**control**	60 %	21 %	16 %	3.1 %	64 %	22 %	12 %	2.1 %
