# Supplementary figures and images for: Safety Studies of Pneumococcal Endolysins Cpl-1 and Pal
Source: Viruses. 2018 Nov 15;10(11):638. doi: 10.3390/v10110638 (PMC6266847; doi:10.3390/v10110638)

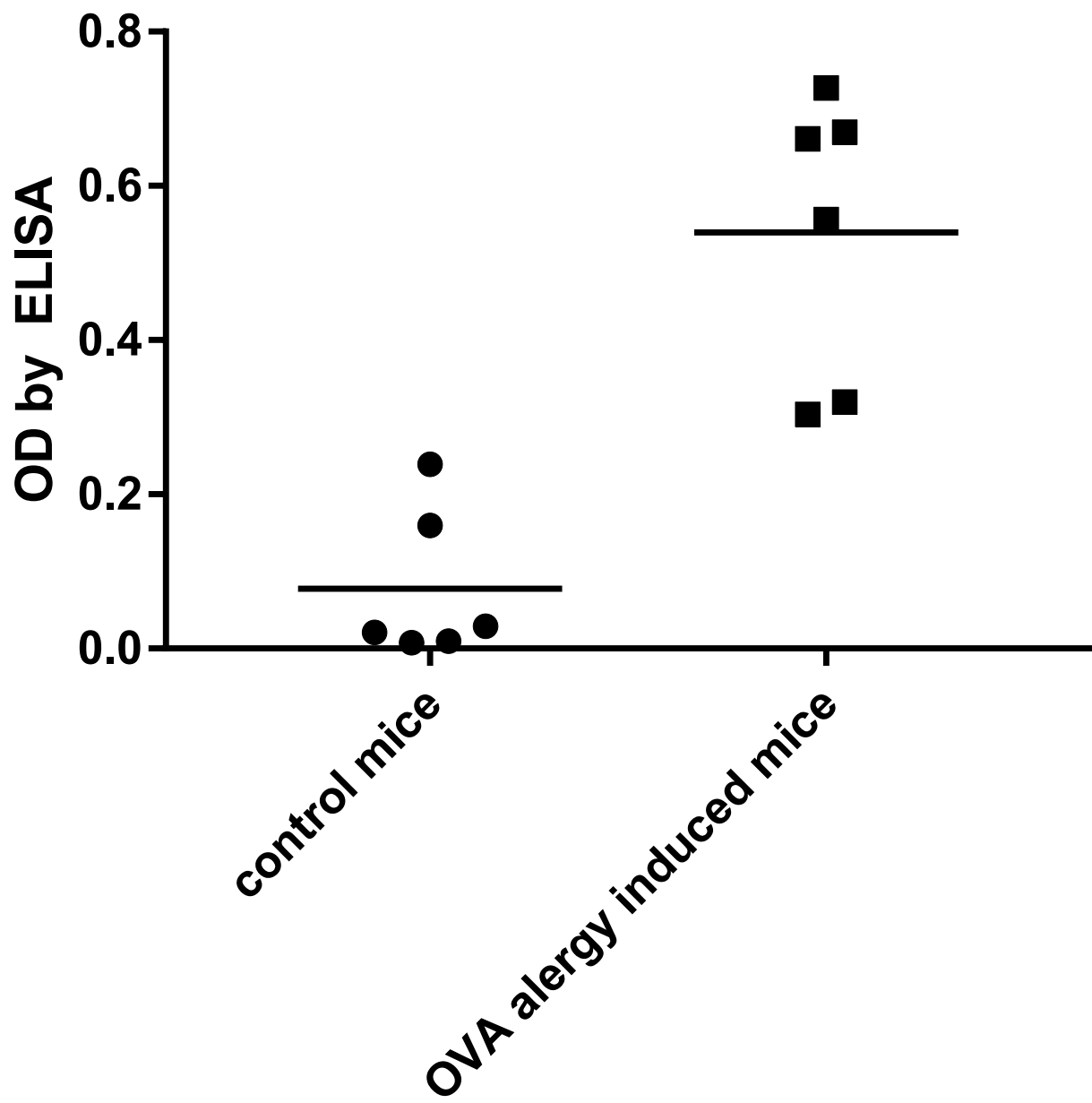

Supplement: Supplementary file 1 [file viruses-10-00638-s001.zip › Supplementary/Figure_S3.pdf]
